# Genome-Wide Analysis of the Histone Modification Gene (*HM*) Family and Expression Investigation during Anther Development in Rice (*Oryza sativa* L.)

**DOI:** 10.3390/plants13172496

**Published:** 2024-09-06

**Authors:** Yongxiang Huang, Jiawei Liu, Long Cheng, Duo Xu, Sijia Liu, Hanqiao Hu, Yu Ling, Rongchao Yang, Yueqin Zhang

**Affiliations:** College of Coastal Agricultural Sciences, Guangdong Ocean University, Zhanjiang 524088, China

**Keywords:** rice (*Oryza sativa* L.), histone modification gene family, genome-wide, anther development, expression analysis

## Abstract

Histone modification plays a crucial role in chromatin remodeling and regulating gene expression, and participates in various biological processes, including plant development and responses to stress. Several gene families related to histone modification have been reported in various plant species. However, the identification of members and their functions in the rice (*Oryza sativa* L.) *histone modification* gene family (*OsHM*) at the whole-genome level remains unclear. In this study, a total of 130 *OsHMs* were identified through a genome-wide analysis. The *OsHM* gene family can be classified into 11 subfamilies based on a phylogenetic analysis. An analysis of the genes structures and conserved motifs indicates that members of each subfamily share specific conserved protein structures, suggesting their potential conserved functions. Molecular evolutionary analysis reveals that a significant number of *OsHMs* proteins originated from gene duplication events, particularly segmental duplications. Additionally, transcriptome analysis demonstrates that *OsHMs* are widely expressed in various tissues of rice and are responsive to multiple abiotic stresses. Fourteen *OsHMs* exhibit high expression in rice anthers and peaked at different pollen developmental stages. RT-qPCR results further elucidate the expression patterns of these 14 *OsHMs* during different developmental stages of anthers, highlighting their high expression during the meiosis and tetrad stages, as well as in the late stage of pollen development. Remarkably, *OsSDG713* and *OsSDG727* were further identified to be nucleus-localized. This study provides a fundamental framework for further exploring the gene functions of *HMs* in plants, particularly for researching their functions and potential applications in rice anthers’ development and male sterility.

## 1. Introduction

*Histone modifier* gene families (*HMs*) play a crucial role in regulating plant growth and development by modulating gene expression through histone modification processes, including methylation, demethylation, acetylation, and deacetylation. These distinct histone modification processes are governed by four unique members of the histone modification gene family: histone methyltransferases (*HMTs*), histone demethylases (*HDMs*), histone acetyltransferases (*HATs*), and histone deacetylases (*HDACs*) [1].

Histone methylation is a mechanism that modulates chromatin structure, playing a pivotal role in the regulation of gene expression in both animals and plants. This modification can impact nucleosome quantity and influence their interactions with other proteins, especially in gene transcription regulation. The methylation of histones can either enhance or suppress gene transcription, a process highly dependent on the specific amino acids that undergo methylation and the number of methyl groups added. Notably, histones can solely be methylated on lysine (K) and arginine (R) residues, with lysine residues in histone tails H3 and H4 being the most common sites for methylation [2]. Histone methylation plays a critical role in a range of plant physiological processes, including their development, adaptation to environmental stresses, and the maintenance of genome stability [3,4,5]. *SDG714*, a histone H3K9 methyltransferase in rice (*Oryza sativa* L.), silences transposable elements like *Tos17* by maintaining DNA methylation, thereby ensuring genome stability [6]. On the contrary, histone methylation can also be directly reversed through the activity of histone demethylases [4]. In rice, the overexpression of *OsJMJ703* resulted in changes to panicle morphology, while its downregulation is associated with earlier flowering and improved drought tolerance [7]. *OsJMJ718*, a rice gene involved in H3K9 methylation, is critical for seed germination and vigor through abscisic acid (ABA) and ethylene signaling pathways [8].

Meanwhile, histone acetylation and deacetylation represent another mechanism that dynamically governs chromatin’s structure and transcriptional regulation [9,10]. The equilibrium of histone acetylation is upheld by two classes of opposing proteins: histone acetyltransferases and histone deacetylases. Genes encoding HATs have been extensively reported for regulating developmental transitions, responding to environmental cues, and integrating signals from stress hormones [11]. Genetic and physiological studies have demonstrated that plant *HDACs* assume significant roles in diverse biological processes, encompassing seed germination, organ development, flowering, responses to both biotic and abiotic stresses, and leaf senescence [12,13,14]. For instance, *HDA6*, a member of the RPD3/HDA1 family proteins, plays a role in jasmonic acid-mediated plant defense responses, senescence, and flowering [15]. *HDT701*, a histone deacetylase in rice, inhibits seed germination by repressing gibberellin biosynthetic genes and enhancing seedling tolerance to salt and osmotic stress [16]. *OsHDA716* reduces cold tolerance by deacetylating OsbZIP46 to decrease its activity and stability in rice [17].

Throughout the life cycle of flowering plants, the well-development of anthers and the formation of viable pollen are crucial. They represent a fundamental guarantee for the successful accomplishment of double fertilization and the completion of generational reproduction in flowering plants [18]. During anther development, epigenetic modifications of histones, including histone methylation and demethylation and histone acetylation and deacetylation, play a significant role in regulating the transcriptional control of genes associated with anther development [19,20]. These modifications facilitate the dynamic and orderly transcriptional regulation of genes involved in anther development [21]. *ASHR3* is the first *SDG* gene found to be associated with male sterility in Arabidopsis. *The ashr3* mutant exhibits normal pollen morphology, including well-formed nutrient nuclei and sperm cells, but the pollen tubes cannot elongate, resulting in a large number of sterile ovules. Furthermore, the mutant displays a complete loss of covalent modification markers for demethylated H3K4 and trimethylated H3K36 in the mature pollen trophoblast nucleus, which ultimately leads to male sterility [22]. Similarly, mutations in *ASHH2*, another *SDG* gene, results in the abortion of approximately 90% of pollen grains with the abnormal phenotype evident from the pollen mother cell stage, with defects in tetrad formation during meiosis [23]. Studies also indicate that overexpression of *ATXR6*, driven by its native promoter, results in impairing anther dehiscence and leads to male sterility in transgenic plants [24,25].

Rice (*Oryza sativa* L.) is a crucial staple crop, providing dietary carbohydrates for over half of the global population, and it is a model organism for monocotyledonous crop research. Rice is widely cultivated in Asia and Africa. Understanding the regulatory mechanisms controlling key traits such as yield, quality, stress resistance, and fertility is crucial for enhancing rice yield and ensuring global food security. Epigenetic mechanisms, particularly DNA methylation and histone modifications, play crucial regulatory roles in these traits. And the emergence of high-throughput sequencing technology has greatly accelerated species genomics research, facilitating a genome-wide analysis. Through comprehensive genome-level analyses across different plant species, variations in the numbers of *HM* gene family members have been observed. For example, oil palm (*Elaeis guineensis* Jacq.) contains 109 *HMs* [26], apple (*Malus pumila* Mill.) boasts a total of 198 *HMs* [27], tomato (*Solanum lycopersicum* L.) harbors 125 *HMs* [28], oranges (*Citrus sinensis* [L.] Osbeck) possess 136 *HMs* [29], grapes (*Vitis vinifera* L.) feature 117 *HMs* [30], and litchi (*Litchi chinensis* Sonn. cv. Feizixiao) include 87 *HMs* [31]. However, there have been no published genome-wide investigations on the *HM* gene family members in the rice genome to date.

In this study, a total of 130 *OsHM* gene family members were identified through a comprehensive genome-wide analysis. This analysis included the examination of their chromosomal locations, gene structures, conserved motifs, gene duplication events, phylogenetic relationships, and cis-elements composition. Additionally, this study conducted an in-depth analysis of their tissue-specific expression patterns and responses to abiotic stresses. Furthermore, the expression patterns of family members at different stages of rice anther development were explored using RT-qPCR. This research shed light on their potential biological functions, especially in the regulation of the development of rice anthers.

## 2. Results

### 2.1. Characterization of the HM Gene Family in the Rice Genome

In this study, we identified a total of 130 *OsHM* gene family members through a comprehensive analysis of the Nipponbare rice genome. This includes 55 histone methyltransferases (*HMTs*), 23 histone demethylases (*HDMs*), 32 histone acetylases (*HATs*), and 20 histone deacetylases (*HDACs*). Based on their protein domain structures, all *OsHM* members can be classified into 11 subfamilies, namely, *SDG*, *PRMT*, *HDMA*, *JMJ*, *HAG*, *HAM*, *HAM*, *HAC*, *HAF*, *HAD*, *SRT*, and *HDT*. The *HMT* family comprises 47 *SDGs* and 8 *PRMTs*, the *HDM* family consists of 9 *HDMAs* and 14 *JMJs*, the *HAT* family includes 26 *HAGs*, 2 *HAMs*, 3 *HACs*, and 1 *HAF*, while the *HDAC* family encompasses 16 *HDAs*, 2 *SRTs*, and 2 *HDTs* (Table 1). All of the gene IDs of *OsHM* gene family members were provided in Table S3.

### 2.2. Chromosome Distribution of OsHM Members in the Rice Genome

**Figure 1:**
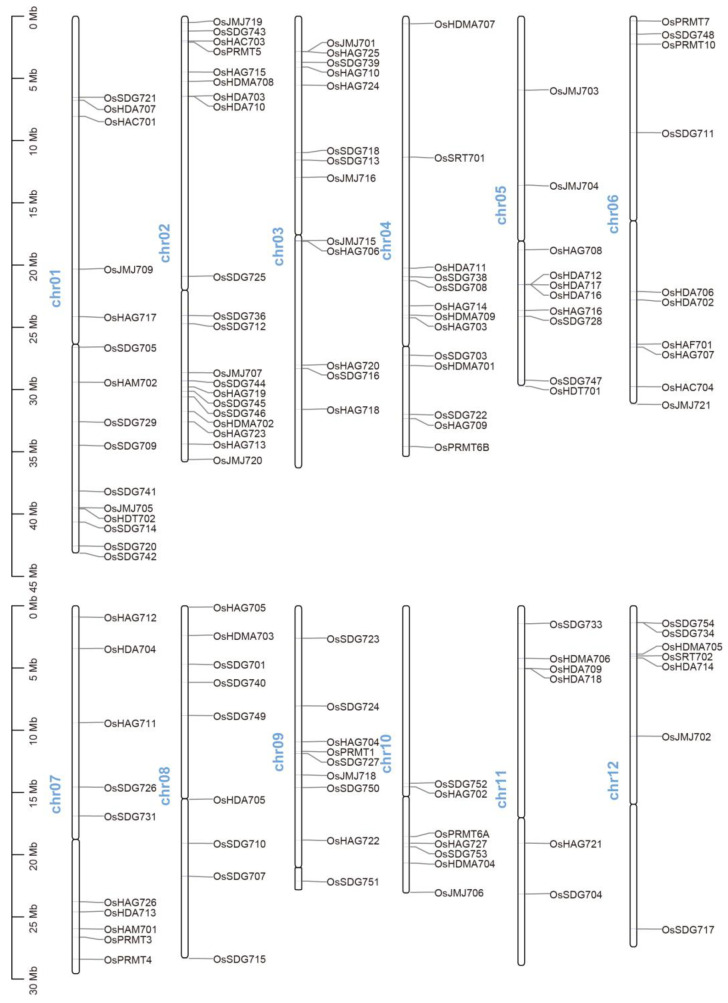
Chromosomal distribution of *OsHM* genes across 12 chromosomes of rice (*Oryza sativa* L.) genome. Chromosome numbers are indicated on the left side of each chromosome. The vertical greyscale on the left side represents the length of the rice chromosomes.

The chromosomal distribution of the 130 *OsHM* members was systematically examined across the 12 chromosomes of the rice genome. Notably, the distribution of *OsHM* gene family members on the chromosomes exhibited a relatively uniform pattern, albeit with varying numbers of members present on all 12 chromosomes. Specifically, chromosome 2 harbors the highest number of *OsHM* gene family members (20 members), while chromosome 11 contains only 6 *OsHM* gene family members (Figure 1). Apart from chromosome 11, all other chromosomes carry at least seven or more *OsHM* gene family members for each. In addition to their chromosomal distribution, *OsSDG705*, *OsJMJ715*, *OsHAG706*, and *OsHDA705* are located near the centromere, while *OsSDG715*, *OsSDG742*, *OsJMJ706*, *OsJMJ720*, *OsJMJ721*, *OsHDT701*, and *OsHAG705* are positioned near the telomere. These genes, situated in specialized regions, are probably involved in maintaining telomere integrity, ensuring correct chromosome segregation, and promoting chromosomal stability.

### 2.3. Gene Structure, Conserved Motifs, and Phylogenetic Analysis of OsHM Gene Family Members

**Figure 2:**
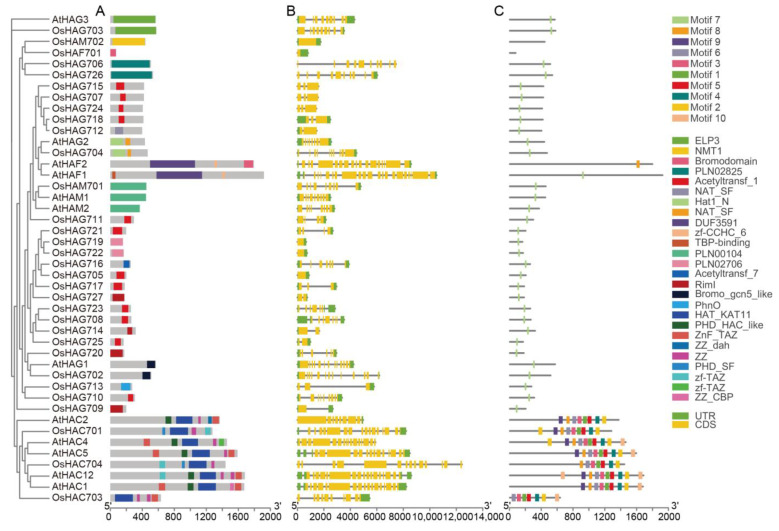
Phylogenetic tree, SMART protein structure prediction (**A**), gene structure (**B**), and motif analysis (**C**) of the *HAT* family in Arabidopsis and rice. Coding sequences (CDS) are represented by yellow color blocks; 3′ and 5′ UTRs are regions represented by green color blocks; and intron regions are represented by gray color blocks in the diagram.

**Figure 3:**
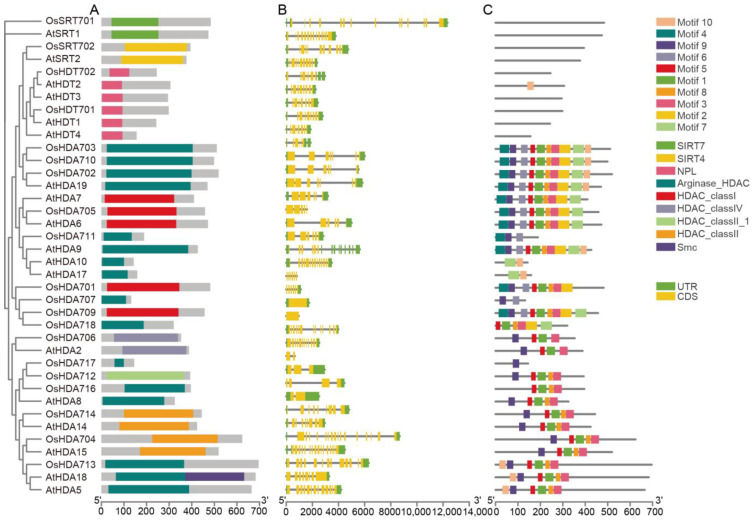
Phylogenetic tree, SMART protein structure prediction (**A**), gene structure (**B**), and motif analysis (**C**) of the *HDAC* family in Arabidopsis and rice. Coding sequences (CDS) are represented by yellow color blocks; 3′ and 5′ UTRs are regions represented by green color blocks; and intron regions are represented by gray color blocks in the diagram.

**Figure 4:**
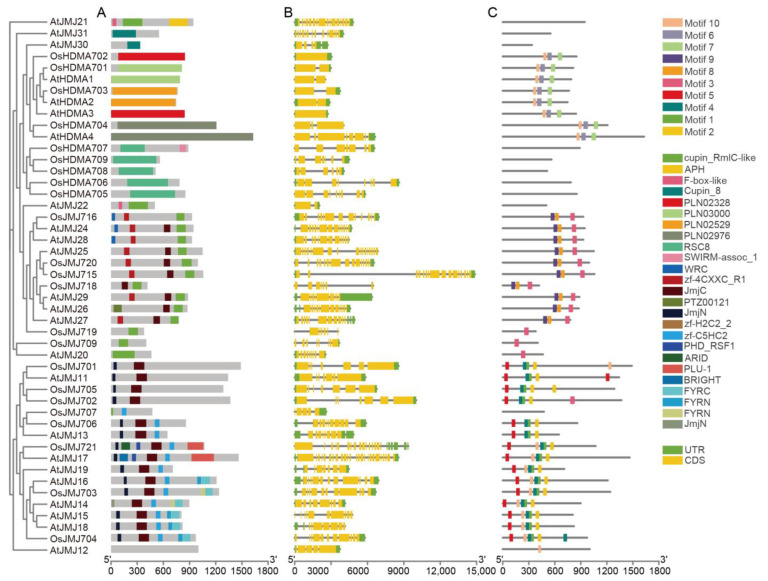
Phylogenetic tree, SMART protein structure prediction (**A**), gene structure (**B**), and motif analysis (**C**) of the *HDM* family in Arabidopsis and rice. Coding sequences (CDS) are represented by yellow color blocks; 3′ and 5′ UTRs are regions represented by green color blocks; and intron regions are represented by gray color blocks in the diagram.

**Figure 5:**
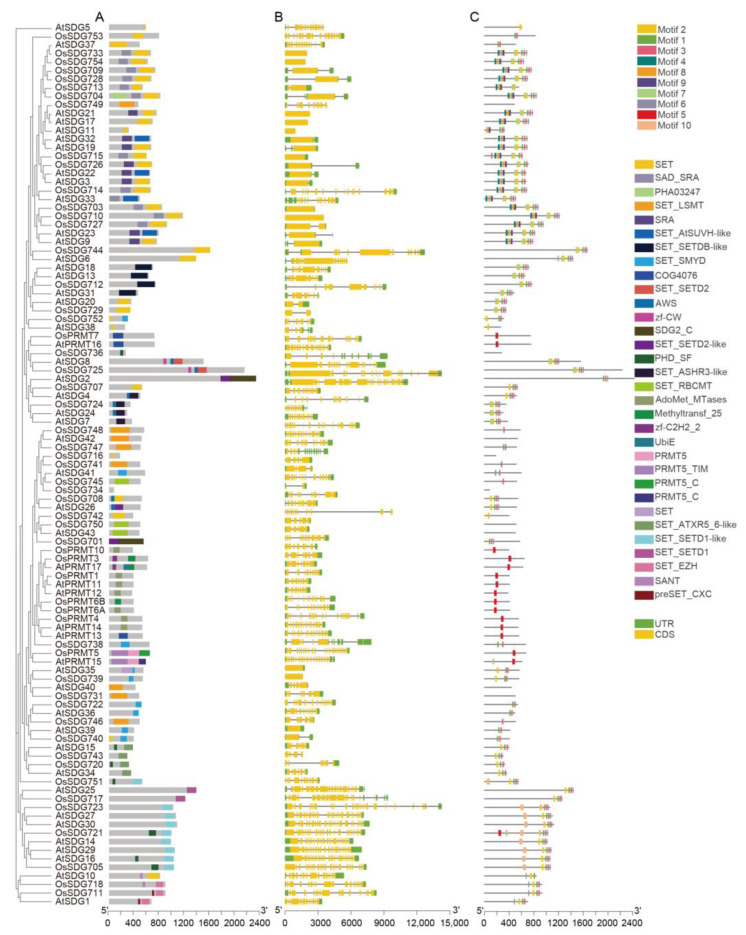
Phylogenetic tree, SMART protein structure prediction (**A**), gene structure (**B**), and motif analysis (**C**) of the *HMT* family in Arabidopsis and rice. Coding sequences (CDS) are represented by yellow color blocks; 3′ and 5′ UTRs are regions represented by green color blocks; and intron regions are represented by gray color blocks in the diagram.

A Gene Structure Display Server 2.0 (http://gsds.cbi.pku.edu.cn/ accessed on 30 July 2024) was employed to analyze the gene structures of all the *OsHM* gene family members. The results revealed variations in the number of exons among the *OsHM* gene family members. *OsHAG705*, *OsHAG719*, *OsHAG722*, *OsHAM702*, *OsHAF701*, *OsHDA709*, *OsHDA718*, *OsHDMA702*, *OsSDG739*, *OsSDG703*, *OsSDG710*, *OsSDG715*, and *OsSDG733* displayed the lowest number of exons, with just 1, while *OsSDG723* exhibited the highest number of exons, with 25 (Figure 2, Figure 3, Figure 4 and Figure 5B).

Motif analysis revealed that ten conserved motifs were identified among the 130 members of the *OsHM* gene family (Figure 2, Figure 3, Figure 4 and Figure 5C). To elucidate the evolutionary relationships among the *HM* gene family members of rice, phylogenetic trees were constructed and analyzed for each *HM* gene family, including *HATs*, *HDACs*, *HDMs*, and *HMTs*. In the case of the *HAT* gene family, it was observed that all *HAGs*, *HAMs*, and *HAFs* from rice and Arabidopsis did not cluster together. Instead, they displayed species-specific or mixed clustering patterns. Conversely, *HACs* clustered well together. A domain composition analysis indicated that within the HAG family, *OsHAG703*, *OsHAG702*, and *OsHAG704* belong to the ELP3, GCN5, and HAT1 classes, respectively, while the other members contain at least the AT1 conserved domain. As for *OsHAM701*, it consists of the MOZ_SAS motif (PF01853). The domain composition of the three *OsHACs* is similar, with *OsHAC703* containing an additional ZnF_TAZ domain (Figure 2A).

For the *HDAC* gene family, *HDTs*, *SRTs*, and *HDAs* clustered within the same subfamily, being classified into three categories, each of which contains the conserved Hist_deacetyl domain (PF00850) and an additional STYKc domain (SM00221). Two *OsSRTs* possess the SIR2 domain (PF02146), while *OsSRT701* harbors a SIRT7 domain, and *OsSRT702* contains a SIRT4 domain (Figure 3A). A phylogenetic tree analysis of HDT showed that *OsHDT701* is closely related to *AtHDT1*, while *OsHDT702* is most closely to *AtHDT2* and *AtHDT3* (Figure 3A).

The phylogenetic tree for *HDMs* also exhibited distinct patterns, with *AtJMJ30* clustering together with *HDMAs*, while the remaining genes clustered in a subfamily specific manner. A conserved domain analysis showed that all *OsHDMAs* contain the N-terminal SWIRM domain (PF04433) and C-terminal Amino oxidase domain (PF01593). The JMJ family is divided into five categories based on sequence similarity, including pure JMJ, KDM3, KDM4, KDM5, and JMJD6. *OsJMJ709* belongs to the pure JMJ category, which only contains the JmjC domain and is not assigned to any other class. *OsJMJ715*, *OsJMJ716*, *OsJMJ718*, *OsJMJ719*, and *OsJMJ720* belong to the KDM3 category, characterized by the presence of a JmjC domain at the C-terminus and a Tudor domain (SM000184) at the N-terminus (Figure 4A). The KDM4 category is divided into two major subclasses based on the composition of protein domains. *OsJMJ701*, *OsJMJ702*, and *OsJMJ705* belongs to subclass I, which is characterized by four tandem ZnF_C2H2 domains (SM000355). And *OsJMJ706* and *OsJMJ707* belong to subclass II, which contains a zf-C5HC2 domain (PF02928) at the C-terminus. In addition, *OsJMJ703* and *OsJMJ704* belong to the KDM5 category (Figure 4A).

In the case of the *HMT* gene family, it was evident that the majority of *SDGs* and *PRMTs* genes clustered together, except for *OsSDG738* and *PRMTs*, which clustered together, and *OsPRMT7* and *AtPRMT16*, which clustered with *SDGs*. Conserved domain analysis indicated that all *OsSDGs* can be classified into five categories. In detail, *OsSDG711* and *OsSDG718* cluster together with two *AtSDGs* and belong to class I (Figure 5A). Class II consists of four ASH1-like *OsSDGs* that cluster with five Arabidopsis ASH1-like proteins. These *OsSDGs* contain conserved SET, Post-SET, and AWS domains. The TRX (TRITHORAX) family (class III) includes four *OsSDGs*, characterized with highly conserved SET and post-SET domains. Seven PRMTs and nine *OsSDGs*, containing SET and N-terminal PHD domains, belong to class IV. Thirty-two *OsSDGs* belong to class V, which is further divided into two main clades containing 17 and 15 *OsSDG* members, respectively (Figure 5A).

### 2.4. OsHM Gene Duplication Events in the Rice Genome

**Figure 6:**
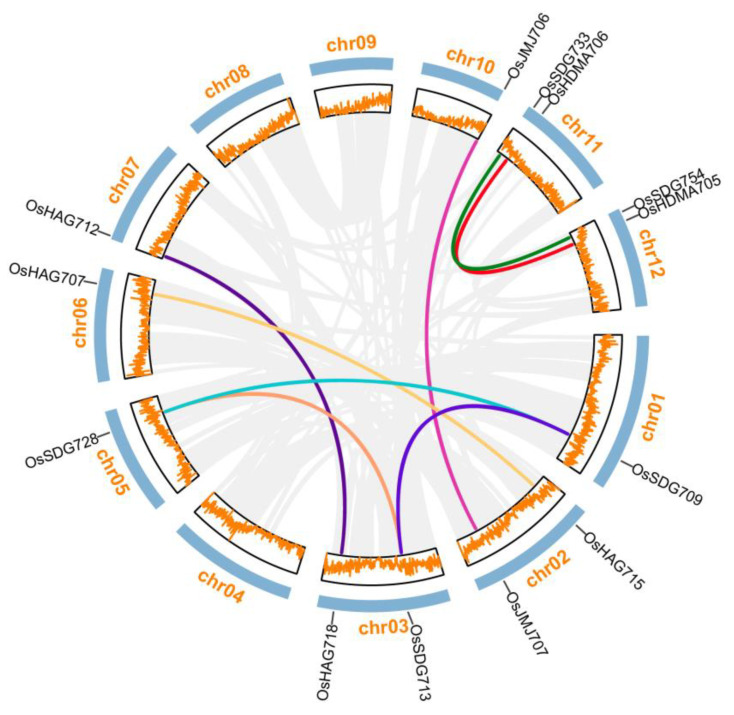
Gene duplication events and interchromosomal relationships between *OsHM* genes in rice genome. A total of eight duplicated *OsHM* gene pairs were found across 12 chromosomes via the MC ScanX tool and are linked by the colorful lines inside the circle view. Grey lines indicate gene duplication events in the rice genome.

To gain a comprehensive understanding of the amplification patterns within the *OsHM* gene family in the rice genome, the study employed the Circos algorithm to generate a gene duplication event diagram. Eight pairs of *OsHMs* were identified across the 12 rice chromosomes, including four pairs of *OsSDGs*, two pairs of *OsHAGs*, one pair of *OsJMJs*, and one pair of *OsHDMAs*. These *OsHMs* occurred in pairs across different chromosomes, with Chromosome 3 harboring an additional three duplicated genes, while Chromosomes 4, 8, and 9 did not exhibit any duplicated *HM* genes (Figure 6). And it is worth to note that all eight pairs of duplicated *OsHM* genes were identified as segmental duplications, which indicates that the expansion of the rice *HM* family is primarily driven by segmental duplications, with the *OsSDGs* subfamily manifesting the most rapid expansion.

Furthermore, to estimate the evolutionary rates of *OsHMs* family duplication events, we calculated the Ka and Ks values for each duplicated gene pair. Among the eight segmental duplications, *OsSDG709* and *OsSDG728* and *OsHDMA706* and *OsHDMA705* displayed similar Ks values, and these gene duplication events occurred approximately 63.2–68.6 million years ago. However, *OsHAG715* and *OsHAG707* and *OsHAG718* and *OsHAG712* displayed similar Ks values, with duplication events around 29.7–31.0 million years ago (Table S4).

To assess selection pressure, we calculated the Ka/Ks ratio, a commonly used indicator for estimating selection pressure on duplicated genes. It is noteworthy that, except for the relatively high sequence divergence between *OsJMJ707* and *OsJMJ706*, the Ka/Ks ratios for the other duplicated events were all below 1. This suggests that these duplicated *HM* genes have undergone a strong purifying selection, preserving the function of ancestral proteins.

### 2.5. Cis-Regulatory Elements Analysis of OsHMs

**Figure 7:**
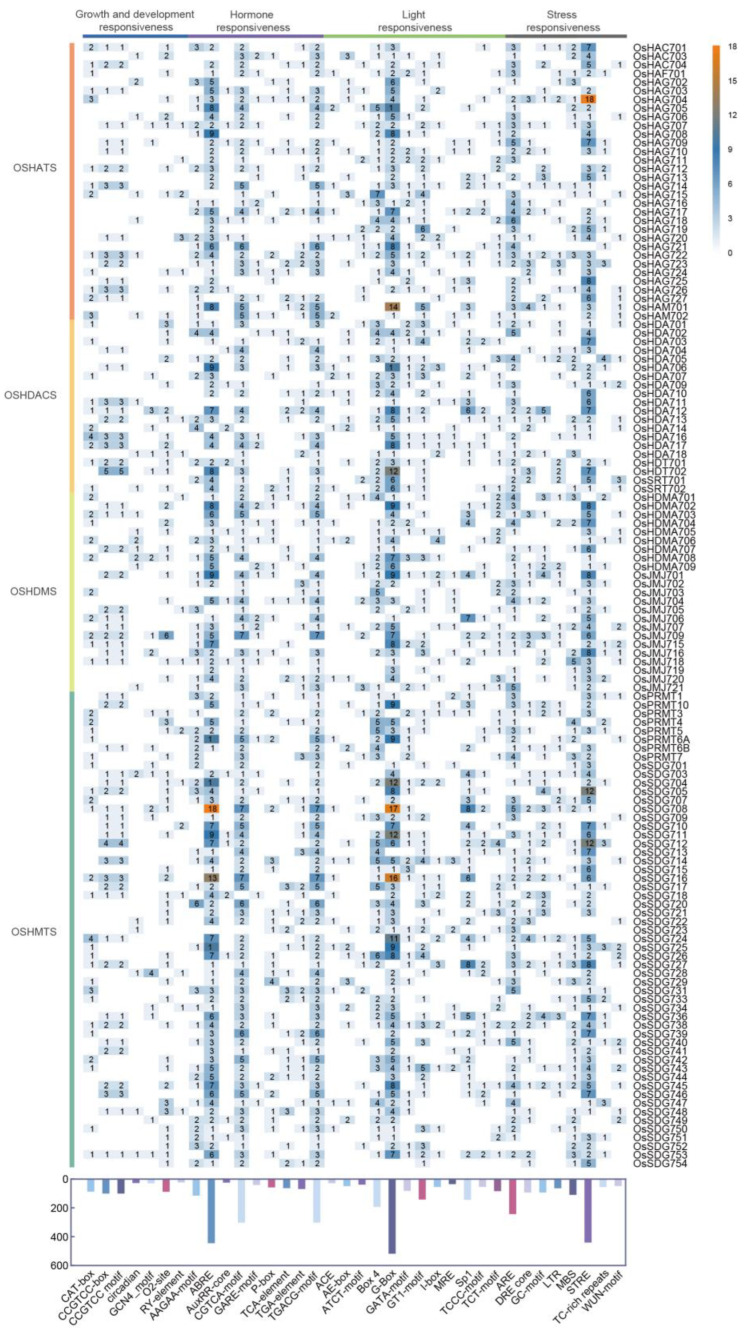
The number of cis-acting elements identified within 2.0 kb promoter regions of *OsHMs* by PlantCARE. The color bar in the upper right indicates the number of cis-acting elements, with orange color representing a higher number of cis-acting elements and blue color representing a low number of cis-acting elements.

Cis-regulatory elements play a crucial role in ensuring the appropriate spatiotemporal expression of genes, which is essential for the proper development and response to the environment in plants. And their divergence is a common cause of evolutionary change, conferring morphological variation and environmental acclimation [32,33]. To analyze the cis-regulatory elements of *OsHMs* in-depth, we obtained 2.0 kb of the promoter regions of all of the 130 *OsHMs* and conducted an analysis of cis-elements using PlantCARE. We identified 36 types of cis elements, categorized into four main classes, including those involved in plant hormones responses (auxin, gibberellin, MeJA, salicylic acid, and abscisic acid), plant growth and development responses (endosperm, meristem, zein metabolism, seed, circadian, and cell cycle), light responses, and abiotic stress responses (drought, anaerobic induction, low temperature, and anoxic). The most abundant cis elements identified are associated with light responses and plant hormones responses, with 1412 and 1411, respectively. Notably, the most numbers of cis-elements are related to MeJA and abscisic acid hormone responses, with 602 and 444, respectively. These findings imply that *OsHM* genes probably play roles in plant growth, hormone signaling, and abiotic stress responses, particularly in light response, MeJA, and abscisic acid signaling pathways (Figure 7).

### 2.6. Tissue-Specific Expression Pattern Analysis of OsHMs in Rice

To analyze the tissue-specific expression patterns of the *OsHM* gene family members in rice, a search in the Rice Genome Annotation Project database was conducted and obtained 103 *OsHMs* genes with expression data (FPKM values) in various rice tissues, including 20 d leaves, emerging inflorescence, early inflorescence, anthers, pistils, seeds at 5 and 10 days after pollination, embryos at 25 days after pollination, and endosperm at 25 days after pollination. Heatmaps were generated using available FPKM values to examine the transcriptional abundance of *OsHMs* at different developmental stages in rice.

**Figure 8:**
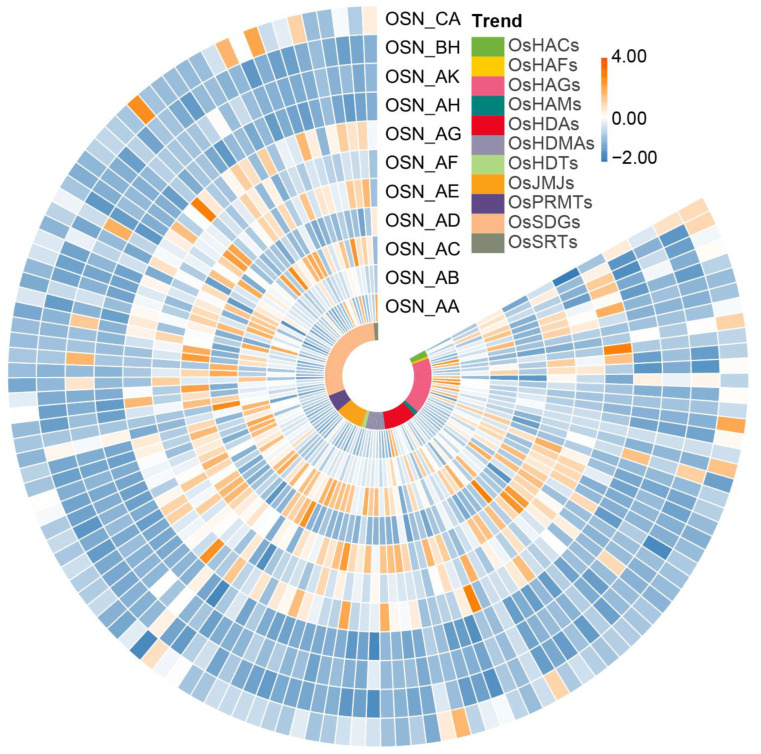
Expression levels of the *HM* family genes in rice at different tissue sites in rice. OSN_AA: 20 d leaves, OSN_ AB: emerging inflorescence, OSN_AC: early inflorescence, OSN_AD: anther, OSN_AE: pistil, OSN_AF: seed 5 d after pollination, OSN_AG: embryo 25 d after pollination, OSN_AH: endosperm 25 d after pollination, OSN_AK: seed 10 d after pollination, OSN_BH: endosperm 25 d after pollination, OSN_CA: 20 d leaves. The color bar in the top right corner represents the Log2FC values for the expression levels of *OsHMs* family genes, with orange indicating high expression and blue indicating low expression.

As depicted in Figure 8, *OsHM* gene family members exhibited varying expression levels in different tissues of rice, with some exclusively expressed in specific tissues. For example, *OsHAT* and *OsHDAC* subfamily members showed higher expression levels in 20 d leaves, early inflorescences, and anthers, while *OsHDM* and *OsHMT* subfamily members displayed higher expression levels in early inflorescences and pistils. In contrast, most of the *OsHM* genes exhibited a low expression level in emerging inflorescence, endosperm at 25 d after pollination, and seeds at 10 d after pollination. In the case of seeds at 5 d after pollination, highly expressed members were concentrated primary in the *OsHATs* and *OsHMTs* subfamilies. Notably, most genes were lowly expressed in anthers, but six members of *OsHMTs* (*OsSDG707*, *OsSDG712*, *OsSDG713*, *OsSDG720*, *OsSDG723*, *OsSDG727*), two members of *OsHDMs* (*OsJMJ704*, *OsJMJ705*), four members of *OsHATs* (*OsHAG707*, *OsHAG714*, *OsHAG722*, *OsHAG724*), and two members of *OsHDACs* (*OsHDA701*, *OsSRT702*) were found to be highly expressed during the anther development (Figure 8). Among these 14 *OsHM* genes, except for *OsHAG707*, *OsHAG714*, and *OsSRT702*, all other *OsHMs* exhibited anther-specific expression patterns. For example, *OsHDA701* displayed expression exclusively in anthers, while *OsSDG713* exhibited expression only in anthers and seeds at 10 days after pollination. In summary, our findings suggest that *OsHATs* and *OsHMTs* probably play pivotal regulatory roles in rice anther development.

### 2.7. Abiotic Stress Response Analysis of OsHMs in Rice

**Figure 9:**
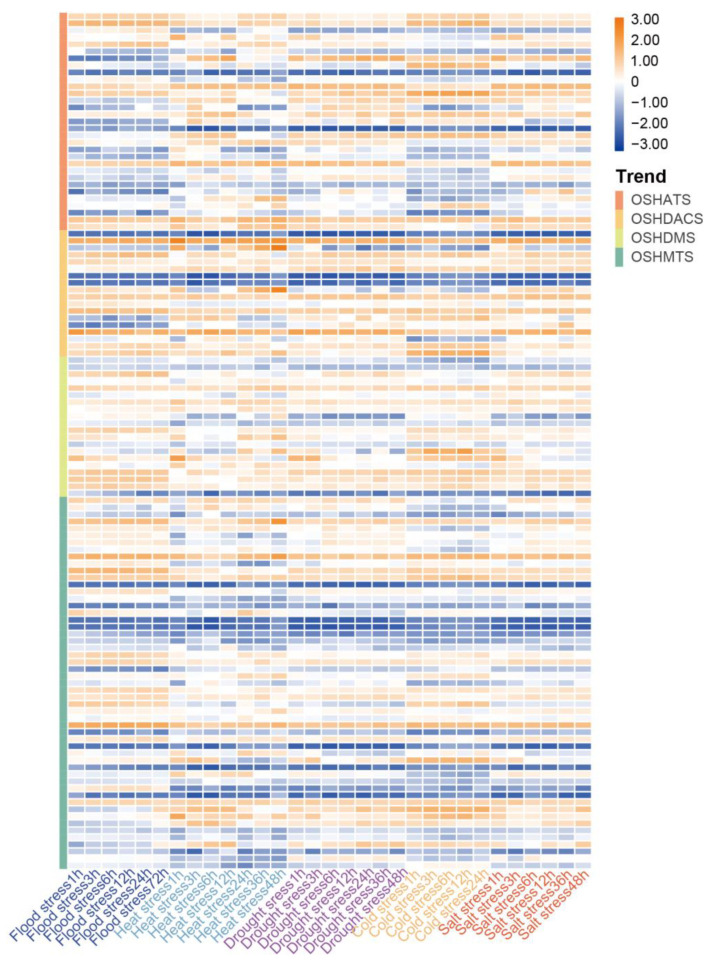
Expression analysis of *OsHMs* in response to various abiotic stress conditions, including flooding stress, heat stress, drought stress, cold stress, and salt stress. The vertical bands on the left side indicate the different subfamilies of rice. The color bar in the top right corner represents the Log2FC values for the expression levels of the *OsHM* family genes, with orange indicating high expression and blue indicating low expression.

To further investigate the response of the *OsHM* gene family members to abiotic stresses, expression data for 122 *OsHM* gene family members under various abiotic stresses, including flooding stress, heat stress, drought stress, cold stress, and salt stress, were obtained from the Rice RNA-seq database. The analysis revealed members with significantly upregulated expression under abiotic stress spanned all of the four subfamilies. Specifically, four members of *OsHMTs* (*OsSDG701*, *OsSDG705*, *OsSDG728*, *OsSDG744*), two members of *OsHDMs* (*OsHDMA706*, *OsJMJ708*), two members of *OsHATs* (*OsHAC703*, *OsHAM701*), and two members of *OsHDACs* (*OsHDA702*, *OsHDA713*, *OsHDT701*) were significantly up-regulated in response to all five abiotic stresses, indicating their wide involvement in response to abiotic stresses in rice. Under flooding stress, *OsHDA702*, *OsSDG728*, *OsSDG701*, *OsSDG704*, *OsHAC703*, *OsHDA713*, and *OsPRMT4* displayed dynamic expression patterns, especially *OsHDT701*, with the expression being suppressed at 24 h of flooding stress and subsequently recovering after 72 h of flooding stress. And *OsHAG706*, *OsSDG728*, *OsSDG704*, and *OsPRMT4* exhibited gradually increasing expression levels with prolonged flooding stress, peaking at 72 h. Under heat stress, expressions of *OsHDA702* and *OsJMJ707* peaked after 1 h of treatment, while *OsHDA703*, *OsHDA710*, *OsHDA702*, and *OsPRMT4* maintained high expression even after 48 h of heat stress. In response to drought stress, *OsHDT701* and *OsHDA702* consistently exhibited high expression within the initial 24 h of drought, continuing to be highly expressed until 48 h. Additionally, the expression of *OsHAG705* peaked at 24 h of drought stress. Under cold stress, the expression of *OsSDG704*, *OsSDG744*, and *OsJMJ703* was down-regulated, while the expression of *OsSDG720* and *OsSDG721* was up-regulated. Under salt stress, the expression of *OsHAG725* was down-regulated, while *OsHDT701* exhibited a dynamic expression pattern, gradually increasing within the initial 12 h of salt stress, declining after 36 h, and rebounding after 48 h (Figure 9). These findings indicate that *OsHM*s probably play important roles in response to a wide range of abiotic stresses, and different *OsHM*s members are required for different abiotic stress in rice.

### 2.8. Expression Pattern Analysis of OsHMs in Rice Anthers Development

**Figure 10:**
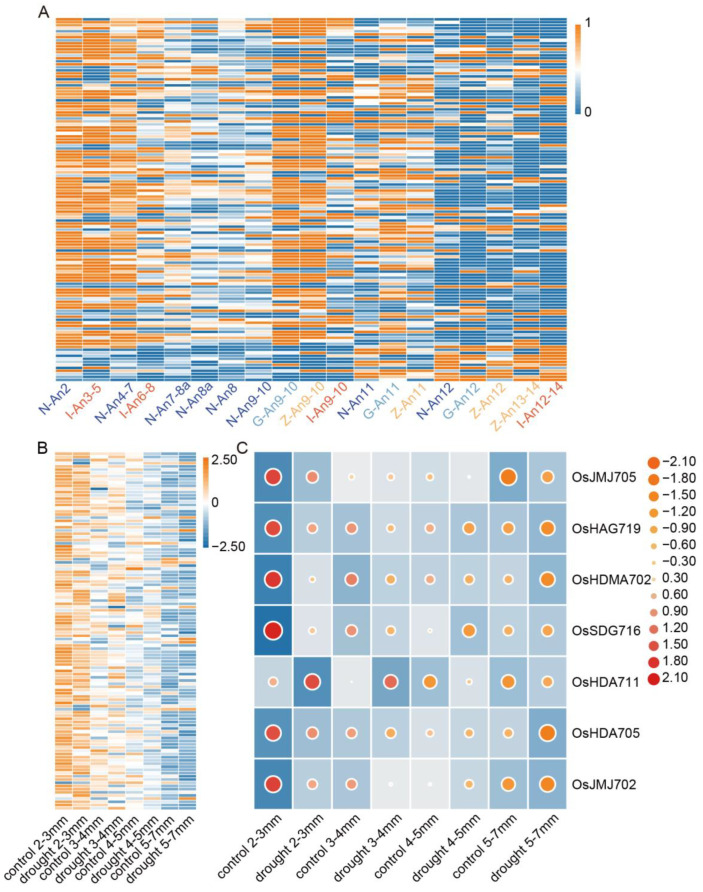
Expression levels of *OsHMs* at different developmental periods of rice anthers. (**A**) Nipponbare anther developmental stage 2–12; IR64 anther developmental stage 3–14; Zhonghua 10 anther developmental stage 9–14; Guichao No. 2 anther developmental stage 9–12. The color bar in the top right corner represents the Log2FC values for the expression levels of *OsHMs* family genes, with orange indicating high expression and blue indicating low expression. (**B**) Response of *OsHM* gene family members to anther drought stress; (**C**) expression analysis of the seven key drought stress-response genes. The color dot in the right represents the Log2FC values for the expression levels of *OsHMs* genes, with red indicating high expression and orange indicating low expression.

Analysis of gene expression profiling can provide valuable clues for predicting their functions in plant development and responses to environmental stimuli [34,35]. To investigate the potential roles of *OsHMs* in rice anther development, we conducted expression profiling analysis on anthers at different developmental stages using the available RNA-seq data. The transcriptome data of four rice varieties with different anthers developmental stages, including Nipponbare anthers at stages 2, 4–7, 7–8a, 8a, 8, 10, 11, and 12, IR64 anthers at stages 3–5, 6–8, 9–10, and 12–14, Zhonghua10 anthers at stages 9–10, 11, 12, and 13–14, and Guichao NO. 2 anther stages 9–10, 11, and 12 were obtained. The results indicate that *OsHM* gene family members showed relatively higher expression levels at stages 2 and 4–7 of Nipponbare anther development. Similarly, *OsHMs* exhibited high expression levels at stages 3–5 and 6–8 of IR64 anther development. Additionally, results for Zhonghua10 and Guichao No. 2 demonstrated that these family members also displayed relatively higher expression levels at stages 9–10 and 11 of rice anther development. Among the aforementioned 14 *OsHM* genes highly expressed in anthers specifically, *OsSDG723* exhibited high expression at the early stages (stages 2 and 4–7) of Nipponbare anther development. *OsHAG707* showed high expression at the early stages (stages 3–5) of IR64 anther development, and *OsJMJ705* also displayed high expression at stages 2 and 4–7 of Nipponbare anther development and stages 3–5 of IR64 anther development. Together, these results imply that the *OsHMs* may play crucial roles in both the early and late stages of anther development, particularly in early developmental regulation (Figure 10A).

Considering the observed dynamic responses of *OsHM* gene family members to abiotic stress and their stage-specific expression patterns in anther development, we hypothesize that these family members probably are involved in regulating anther adaptability to stress. To address this hypothesis, we further analyzed the expression of *OsHM* gene family members under abiotic stress in anthers. Due to limited transcriptome data related to abiotic stress in anthers, we only obtained expression data of *OsHM* gene family members in anthers under drought stress from the NCBI database. A differential expression analysis revealed that the expression response of *OsHM* members to drought stress varied at different stages of anther development. Specifically, 43, 56, 46, and 48 *OsHM* members were up-regulated at the four distinct developmental stages, respectively. Among them, *OsJMJ705*, *OsHAG719*, *OsHDMA702*, *OsSDG716*, *OsHDA711*, *OsHDA705*, and *OsJMJ702* were identified as drought-responsive genes [36], and these seven genes exhibited transcriptional response to drought stress across all four anther developmental stages. In detail, *OsHDMA702*, *OsSDG716*, and *OsJMJ702* were down-regulated at the 2–3 mm stage. *OsHDA711* was up-regulated at the 3–4 mm stage, *OsHDA705* was down-regulated at the 3–4 mm stage, *OsJMJ705* was up-regulated at the 5–7 mm stage, and *OsHAG719* was down-regulated at the 4–5 mm stage. Notably, *OsHDMA702* displayed a z-score exceeding the twofold standard deviation, indicating a significant response to drought stress. These findings provide evidence that *OsHMs* probably play crucial roles in responding to environmental stress during anther development, and different members can exert stage-specific stress responses (Figure 10B,C).

### 2.9. Expression Analysis of 14 Key Anther Development Genes at Different Anther Development Stages

**Figure 11:**
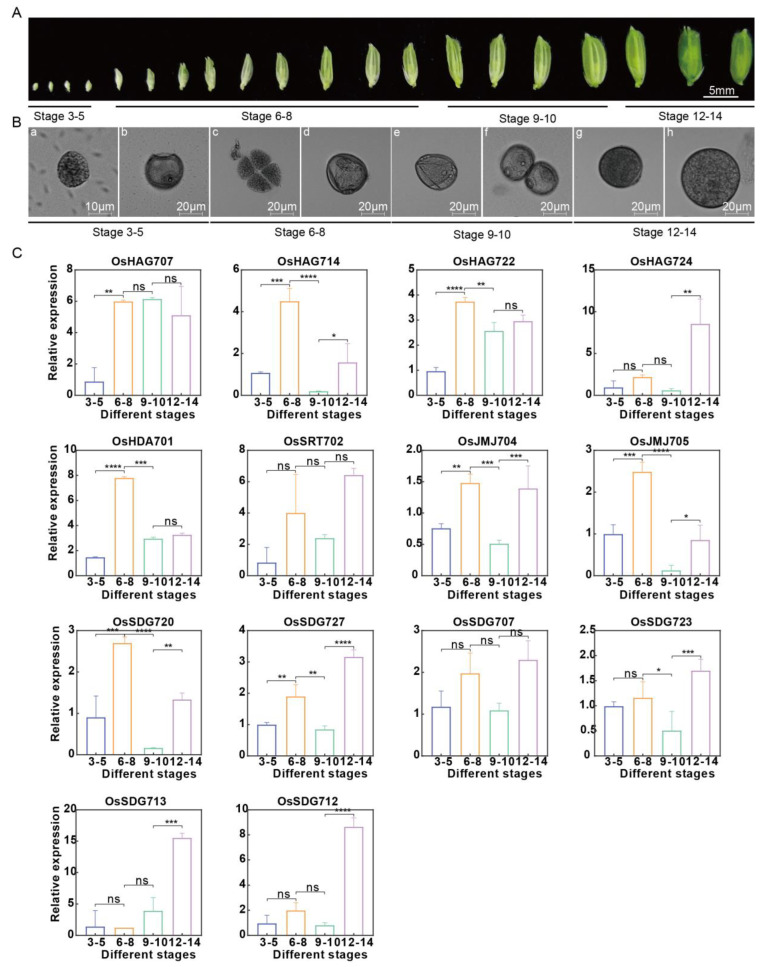
RT-qPCR analysis of *OsHM* genes’ expressions at different anther developmental stages. (**A**) Division of anthers into different developmental periods; (**B**) Photos of anther development at different stages (a-b: the developmental stages of anthers pre-meiosis; c-d: the meiosis to tetrad stages; e-f: the stages of mitosis leading to the formation of binucleate microspores; g-h: the stage of pollen maturity.) taken using an OLYMPUS BX53 microscope. (**C**) Relative expression of *OsHM* genes at different anther developmental stages. Different background color blocks indicate different subfamilies of rice. The *X*-axis represents different stages; the *Y*-axis represents the relative expression of specific *OsHM* gene. Data represents the mean ± SE of three replicates. Asterisks represent significant differences. ns means no significance, *p* ≤ 0.05 (*), *p* ≤ 0.01 (**), *p* ≤ 0.001 (***), and *p* ≤ 0.0001 (****).

According to the cytological morphological differences in anther development, rice anther development is divided into 14 different developmental stages, known as stage1 to stage14 (S1–S14) [37,38]. We further used RT-qPCR to analyze the expression of the 14 *OsHM* genes in detail, which were previously analyzed using RNA-seq data and found to be highly expressed in anthers at different developmental stages of rice anthers. According to Deveshwar’s report, rice anthers of Nipponbare are divided into four different developmental stages based on the floret size. Floret lengths ranging from 1.5 to 2.5 mm correspond to the S3–S5 stages, representing the developmental stages of anthers pre-meiosis. Lengths of 3.5–6 mm correspond to the S6–S8 stages, indicative of the meiosis to tetrad stages. Additionally, lengths of 7–7.5 mm align with the S9–S10 stages, reflecting the stages of mitosis leading to the formation of binucleate microspores. Finally, lengths of 8 mm correspond to the stage of pollen maturity [39]. Accordingly, we selected and microscopically photographed floret samples from four developmental stages, S3–S5, S6–S8, S9–S10, and S12–S14, of anthers from Nipponbare rice (Figure 11A,B). As shown in Figure 11C, RT-qPCR analysis indicated that all of these fourteen *OsHM* genes, including six members of *OsHMTs* (*OsSDG707*, *OsSDG712*, *OsSDG713*, *OsSDG720*, *OsSDG723*, *OsSDG727*), two members of *OsHDMs* (*OsJMJ704*, *OsJMJ705*), four members of *OsHATs* (*OsHAG707*, *OsHAG714*, *OsHAG722*, *OsHAG724*), and two members of *OsHDACs* (*OsHDA701*, *OsSRT702*), were expressed in anthers, which was consistent with the transcriptome results, and they exhibited different expression levels at the different developmental stages of anthers. Most genes were highly expressed during S6–S8 of anther development, corresponding to meiosis and tetrad formation, and during S12–S14, representing pollen maturation. Among them, *OsHAG714*, *OsHAG722*, *OsHDA701*, *OsJMJ704*, *OsJMJ705*, and *OsSDG720* exhibited high expression during S6–S8 of anther development, implying their essential functions during meiosis and tetrad formation. *OsHAG724*, *OsSRT702*, *OsSDG707*, *OsSDG712*, *OsSDG713*, *OsSDG723*, and *OsSDG727* were highly expressed during S12–S14, suggesting their involvement in pollen maturation (Figure 11C).

### 2.10. Subcellular Localization Analysis of OsSDG713 and OsSDG727

**Figure 12:**
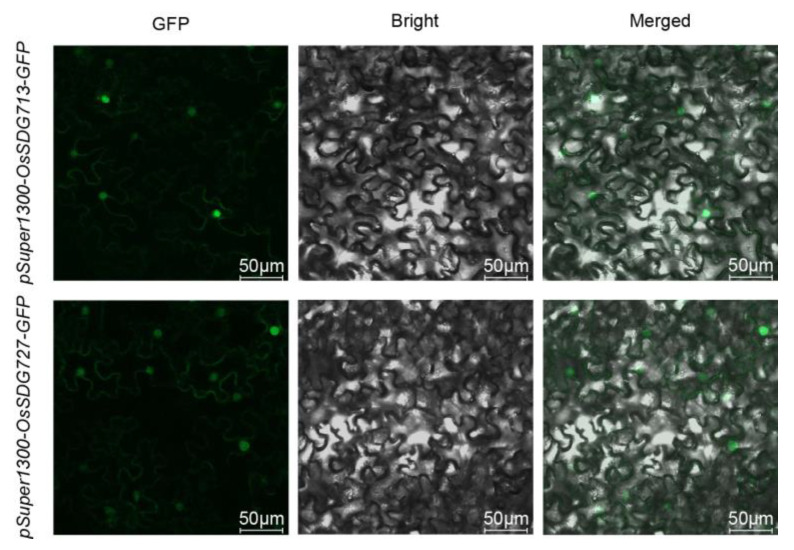
Subcellular localization analysis of OsSDG713 and OsSDG727 in tobacco leaves.

To explore the subcellular localization of OsHM proteins, we selected OsSDG713 and OsSDG727 proteins to analysis due to their potential roles in anther development. We generated *pSuper1300-OsSDG713-GFP* and *pSuper1300-OsSDG727-GFP* vectors and transiently expressed them in tobacco epidermal cells via *Agrobacterium tumefaciens*-mediated infiltration. Confocal microscopy revealed specific nuclear fluorescence signals for both OsSDG713 and OsSDG727, suggesting their nuclear subcellular localization (Figure 12).

## 3. Discussion

In this study, a genome-wide analysis of the *OsHM* gene family was conducted in rice, and we identified a total of 130 *OsHMs* members, which is similar to the numbers of the *HM* gene family observed in other crops, such as apple (198 *HMs*) [27], litchi (87 *HMs*) [31], oil palm (109 *HMs*) [26], tomato (125 *HMs*) [28], and orange (136 HMs) [29]. As reported, the typical domains in the HM gene subfamily are conserved across most crops [40]. Through protein domain analysis, it is evident that *OsHM* members also possess conserved domains similar to those found in the *HM* gene families of other plants, implying their conserved functions. Based on the analysis of conserved domains and phylogenetic relationships, the *OsHM* gene family can be classified into 11 subfamilies, including 55 *OsHMTs* (histone methyltransferases), 23 *OsHDMs* (histone demethylases), 32 *OsHATs* (histone acetylases), and 20 *OsHDACs* (histone deacetylases), which is consistent with the analysis results in other species [22,24]. Among the *HM* gene family, *SET domain group genes* (*SDGs*) exhibit the highest degree of conservation across species. The number of *OsSDGs* in rice is comparable to that of *SlSDGs*, *EgSDGs*, *CsSDGs*, and *AtSDGs* in other species, yet it represents only three-quarters of the *SDG* count in apples (*MdSDGs*). Conversely, *OsHDMAs* are present in quantities two to three times greater than those in *EgHDMAs*, *CsHDMAs*, and *AtHDMAs*, but they account for only half the number of *MdHDMAs*. Additionally, the abundance of *histone acetyltransferase genes* (*HAGs*) varies significantly among species; the number of *OsHAGs* in rice is equal to that of *SlHAGs*, but constitutes only half the count of *MdHAGs* and *CsHAGs*, while being six to seven times more numerous than *AtHAGs* and *EgHAGs*. Furthermore, the numbers of *PRMTs*, *JMJs*, *HAMs*, *HACs*, *HAFs*, *SRTs*, and *HDTs* are relatively consistent across species, with the highest abundance observed in apples. Comparative analysis of these gene family members across different species serves as a valuable tool for investigating the evolution of gene families.

Gene duplication can provide new genetic material for variation and selection, leading to the emergence of specific or novel gene functions [41]. A duplication event analysis revealed that there are eight pairs of duplicated genes within *OsHMs* gene family and all of them are segmental duplication genes, indicating that the expansion of the rice *HM* gene family is primarily driven by segmental duplications. And among these eight pairs of duplicated genes, four of them belong to the *OsSDG* subfamily, which suggest that the *OsSDG* subfamily may play significant roles in the expansion of the *OsHM* gene family.

To well-predict the potential functions of *OsHMs* in rice, a comprehensive analysis of the expression patterns of the *OsHM* gene family in various tissues was conducted through genome-wide studies in this study. The results suggest that *OsHM* members exhibit significant tissue-specific expression properties, particularly the members of *HDAC* subfamily, implying their tissue-specific gene functions. For instance, *OsHDA710* exhibits high expression levels in shoots, seedlings, and stamens, while *OsHDA703* express specifically in callus and sterile seeds, which is consistent with previous research [42]. In Arabidopsis, *HDT1* and *HDT2* have been reported to be involved in root development regulation [43]. Another member of the HDAC subfamily, *HDT3*, is implicated in abscisic acid (ABA) signaling transduction and salt stress response during seed germination through the interaction with histone deacetylase HDA6 and histone H3 [44,45,46].

*HM* genes have been reported to participate in reproductive development regulation, including anther development. For example, *AtSDG4*, the predominant *ASH1-related* gene expressed in pollen, is responsible for histone 3 methylation in inflorescences and pollen grains, and thus participates in pollen tube growth and male fertility formation. An interaction analysis demonstrated that AtSDG4 binds to the ABORTED MICROSPORES transcription factor via the PHD and SET domains, and overexpression of *AtSDG4* leads to stamen defects, anther abortion, and the inhibition of silique growth in Arabidopsis, indicating their crucial roles in anther development [47,48]. *AtSDG2*, another homologous gene, has been reported to participate in the differentiation of anther walls and pollen development in Arabidopsis by mediating the H3K4me3 deposition at *SPOROCYTELESS/NOZZLE* (*SPL*/*NZZ*) and *MALE STILITY1* (*MS1*) for transcriptional activation [49]. Compared with the relatively comprehensive and in-depth studies of *HMs* genes in Arabidopsis, the functions of *HM* genes in rice anther development remain largely unknown. In our study, *OsHDA701* exhibited high expression specifically in anthers. And an RT-qPCR analysis further confirmed the specific expression of *OsHDA701* in the S6–S8 of anther development. These results suggest that *OsHDA701* probably works as crucial regulator in rice anthers development, especially during the meiosis to tetrad stages. This is consistent with the previous study in Kenaf (*Hibiscus Cannabinus* L.), where five *HcHDAC* genes (*HcHDA2*, *HcHDA6*, *HcHDA8*, *HcHDA9*, and *HcSRT2*) were predicted to participate in anther development, with varying high expression levels in anthers [50]. Additionally, *OsSDG707*, the orthologous of *AtSDG4* and *AtSDG2* in rice according to our phylogenetic analysis, exhibits a specific high-expression pattern during the meiosis to tetrad development stages and maturity stages, which strongly imply its potential roles in anther development in rice. Further experiments elucidating their functions and the underlying regulatory mechanisms in rice anther development using molecular, biochemical, and high-throughput sequencing technologies, such as gene editing, transgenics, and protein–protein/DNA interaction analyses, would be instrumental in comprehensively exploring their roles and potential applications in plants.

## 4. Materials and Methods

### 4.1. Identification of OsHMs in the Rice Genome

The members of the *OsHM* gene family were identified through a systematic approach. Initially, the Hidden Markov Model summary file from the pfam database (http://pfam-legacy.xfam.org/ accessed on 30 July 2024) was employed to query published *HM* genes of the *HMT*, *HDM*, *HAT*, and *HDAC* types using their respective pfam IDs. Subsequently, a thorough search for *HM* gene members was conducted in the Rice Genome Database (https://rapdb.dna.affrc.go.jp/ accessed on 30 July 2024) using the HMMER 3.0 tool (http://hmmer.org/ accessed on 30 July 2024). Additionally, known *HM* gene sequences from both Arabidopsis and rice were utilized to further augment the available sequence data for the *OsHM* genes through blast searches within the rice genome database. The coding sequence length of each member within the *HM* gene family was predicted via Blastn searches of the rice genome database. Based on the presence of highly conserved structural domains, the final identification of members within the rice *HM* gene family was achieved, categorizing them into subfamilies including *HMTs* (*SDGs* and *PRMTs*), *HDMs* (*HDMAs* and *JMJs*), *HATs* (*HAGs*, *HAMs*, *HACs*, and *HAFs*), and *HDACs* (*HDAs*, *SRTs*, and *HDTs*).

### 4.2. Gene Structure, Conserved Motif Analysis of OsHMs, and Phylogenetic Relationships of OsHM Genes

Gene structures (introns-exons) of all 130 *OsHM* genes were meticulously analyzed using the Gene Structure Display Server 2.0 (https://gsds.gao-lab.org/Gsds_help.phpavailable at gao-lab.org accessed on 30 July 2024). Furthermore, the conserved motifs within these OsHMs proteins were comprehensively examined utilizing the MEME Suite tool (https://meme-suite.org/meme/ accessed on 30 July 2024). The potential duplication events within the *OsHM* gene family, which comprised 130 members, were systematically investigated in the rice genome using the MCScanX tool (https://github.com/wyp1125/MCScanX accessed on 30 July 2024), employing default parameters. The estimation of duplication event dates utilized the formula T = Ks/2λ. Here, T represents the time since divergence, Ks is the synonymous substitution rate, and λ denotes the clock-like rate of synonymous substitution. For this study, a λ value of 0.65 × 10⁻⁸ was selected based on rice [51]. In parallel, robust phylogenetic trees were skillfully constructed for each *HM* type present in rice, leveraging the MEGA 7.0 (https://www.megasoftware.net/ accessed on 30 July 2024) software and employing the maximum likelihood method. These phylogenetic trees were bootstrapped with 1000 replicates, ensuring the reliability of evolutionary analyses.

### 4.3. Chromosomal Localization of Rice OsHM Genes

The positions of all 130 *OsHM* genes were meticulously mapped onto the 12 chromosomes of rice through a comprehensive search of the rice genome database. Subsequently, a chromosomal localization map was thoughtfully generated utilizing the TBtools-II (https://github.com/CJ-Chen/TBtools-II/releases accessed on 30 July 2024) software.

### 4.4. Cis-Acting Element Analysis

The 2.0 kb upstream sequences start codons of all *OsHM* genes were obtained from the Rice Genome Database and were regarded as *OsHM* gene promoters. The online software PlantCARE (https://bioinformatics.psb.ugent.be/webtools/plantcare/html/ accessed on 30 July 2024)was used for the identification of cis-acting elements. TBtools-II was used for visualizing the distribution of cis-acting elements within the gene promoters. The numbers of identified cis-acting elements of *OsHM* genes are included in Table S1.

### 4.5. Expression Analysis of OsHMs in Different Tissue Parts, Different Anther Development Stages, and Different Stress Conditions in Rice

RNA-seq data from various tissue parts of rice (accessions: OSN_AA: 20 d leaves SRX100741, OSN_ AB: emerging inflorescence SRX100743, OSN_AC: early inflorescence SRX100745, OSN_AD: anther SRX100746, OSN_AE: pistil SRX100747, OSN_AF: seed 5 d after pollination SRX100749, OSN_AG: embryo 25 d after pollination, OSN_AH: endosperm 25 d after pollination SRX100754, OSN_AK: seed 10 d after pollination SRX100755, OSN_BH: endosperm 25 d after pollination SRX100756, OSN_CA: 20 d leaves SRX100757) were systematically retrieved from the NCBI’s Sequence Read Archive (SRA) database (https://www.ncbi.nlm.nih.gov/sra accessed on 30 July 2024). Subsequently, heat maps were skillfully generated using the RPKM values to precisely assess the expression levels of *OsHMs* in diverse tissue sites of rice. For RNA-seq data representing different anther developmental stages, pertinent datasets were diligently obtained from the GEO database (https://www.ncbi.nlm.nih.gov/geo/ accessed on 30 July 2024), specifically: anther developmental stage 2–12 in Nipponbare (GSE14304); anther developmental stage 3–14 in IR64 (GSE27726); anther developmental stage 9–14 in Zhonghua 10 (GSE27988); and Guichao No. 2 anther development stages 9–12 (GSE29080).

### 4.6. RT-qPCR Expression Analysis of OsHM Genes

This study employed Nipponbare rice variety as the test materials. Total RNA was meticulously isolated from 0.5 g rice florets of varying lengths utilizing Trizol reagent (Yeasen, Shanghai, China). Subsequently, 1 μg RNA was used to synthesis the first-strand cDNA using the Hifair^®^ II 1st Strand cDNA Synthesis Kit (gDNA digester plus) (Yeasen, Shanghai, China). qRT-PCR was executed utilizing Hieff^®^ qPCR SYBR Green Master Mix (No Rox) (Yeasen, Shanghai, China), and a CFX96 Touch™ Real-Time PCR System (Bio-Rad) was employed for qRT-PCR, utilizing the subsequent thermocycling conditions: an initial denaturation at 95 °C for 3 min, followed by 40 cycles of denaturation at 95 °C for 15 s, annealing at 60 °C for 15 s, and extension at 72 °C for 15 s. The tomato Actin gene served as the endogenous control for data normalization. qRT-PCR primers were thoughtfully designed based on the cDNA sequence of *OsHMs* genes using Primer 3 (http://primer3.ut.ee/ accessed on 30 July 2024) and are listed in Table S2. Each PCR reaction’s specificity was assessed by analyzing the amplicon’s melting curve. The comparative 2^−ΔΔCT^ method was diligently applied to compute the relative transcript levels in the samples, and each experiment was performed with three technical replicates for every sample.

### 4.7. Plasmid Construction and Subcellular Localization Analysis

The CDS without stop codon of *OsSDG713* and *OsSDG727* were amplified and inserted into the pSuper1300 vector for transient expression. The vector contained a GFP fluorescent tag and the MAS promoter. The resulting plasmids were then introduced into Agrobacterium GV3101 using freeze–thaw methods. Subsequently, either *pSuper1300-OsSDG713-GFP* or *pSuper1300-OsSDG727-GFP* GV3101 strains were transiently introduced into the leaf blades of 5-week-old *N. benthamiana* plants. Protein expression was visualized three days post-injection on the abaxial epidermis of tobacco leaves. Images were captured using a ZEISS LSM800 confocal microscope with ×20 objective. GFP fluorescence was detected using 488 nm laser excitation and 500–540 nm emission filter.

## 5. Conclusions

In this study, a total of 130 *OsHMs* were identified and characterized in rice using a genome-wide analysis. These *OsHMs* were comprehensively analyzed in terms of gene structure, conserved protein domain, chromosomal distribution, molecular phylogenetic relationships, and cis-elements composition. Furthermore, their tissue-specific expression patterns and abiotic stress response expression patterns were analyzed using the RNA-seq data. The expression of 14 *OsHMs* members specifically expressed in anthers were further confirmed using RT-qPCR. Notably, two of these members, *OsSDG713* and *OsSDG727*, were found to be localized within the nucleus, as detected in tobacco epidermal cells. The results of this research serve as a valuable foundation for gaining deeper insights into the functions and practical applications of individual *OsHM* genes in rice in the future.

## Figures and Tables

**Table 1:** Comparison of the number of members of each subfamily of the rice (*Oryza sativa* L.) and Arabidopsis *HM* gene family.

Species	*HAC*	*HAG*	*HAF*	*HAM*	*HDMA*	*JMJ*	*PRMT*	*SDG*	*HDT*	*HDA*	*SRT*
Rice	3	26	1	2	9	14	8	47	2	16	2
Arabidopsis	5	3	2	2	4	20	7	41	4	12	2

## Data Availability

Additional data can be found in supplementary files. RNA-seq data from various tissue parts of rice (accessions: OSN_AA SRX100741, OSN_AB SRX100743, OSN_AC SRX100745, OSN_AD SRX100746, OSN_AE SRX100747, OSN_AF SRX100749, OSN_AG SRX100753, OSN_AH SRX100754, OSN_AK SRX100755, OSN_BH SRX100756, OSN_CA SRX100757) were systematically retrieved from the NCBI’s Sequence Read Archive (SRA) database. Expression data of *OsHM* gene family members under different abiotic stress conditions, including flooding stress, heat stress, drought stress, cold stress, and salt stress were obtained from rice RNA-seq database Zhailab@SUSTech (http://ipf.sustech.edu.cn/pub/ricerna/ accessed on 30 July 2024).

## Supplementary Materials

The following supporting information can be downloaded at: https://www.mdpi.com/article/10.3390/plants13172496/s1, Table S1: The numbers of identified cis-acting elements of *OsHM* genes; Table S2: The specific RT-qPCR primers; Table S3: All of the gene IDs of *OsHM* gene family members; Table S4: Duplication events of the *OsHMs*.
